# Reshaping Plant Biology: Qualitative and Quantitative Descriptors for Plant Morphology

**DOI:** 10.3389/fpls.2017.00117

**Published:** 2017-02-03

**Authors:** Mathilde Balduzzi, Brad M. Binder, Alexander Bucksch, Cynthia Chang, Lilan Hong, Anjali S. Iyer-Pascuzzi, Christophe Pradal, Erin E. Sparks

**Affiliations:** ^1^INRIA, Virtual PlantsMontpellier, France; ^2^Department of Biochemistry and Cellular and Molecular Biology, University of Tennessee-KnoxvilleKnoxville, TN, USA; ^3^Department of Plant Biology, University of GeorgiaAthens, GA, USA; ^4^Warnell School of Forestry and Environmental Resources, University of GeorgiaAthens, GA, USA; ^5^Institute of Bioinformatics, University of GeorgiaAthens, GA, USA; ^6^Division of Biological Sciences, University of Washington-BothellBothell, WA, USA; ^7^Weill Institute for Cell and Molecular Biology and Section of Plant Biology, School of Integrative Plant Sciences, Cornell UniversityIthaca, NY, USA; ^8^Department of Botany and Plant Pathology, Purdue UniversityWest Lafayette, IN, USA; ^9^CIRAD, UMR AGAPMontpellier, France; ^10^Department of Biology, Duke UniversityDurham, NC, USA

**Keywords:** morphology, topology, geometry, leaf, hypocotyl, sepal, roots, ecology

## Abstract

An emerging challenge in plant biology is to develop qualitative and quantitative measures to describe the appearance of plants through the integration of mathematics and biology. A major hurdle in developing these metrics is finding common terminology across fields. In this review, we define approaches for analyzing plant geometry, topology, and shape, and provide examples for how these terms have been and can be applied to plants. In leaf morphological quantifications both geometry and shape have been used to gain insight into leaf function and evolution. For the analysis of cell growth and expansion, we highlight the utility of geometric descriptors for understanding sepal and hypocotyl development. For branched structures, we describe how topology has been applied to quantify root system architecture to lend insight into root function. Lastly, we discuss the importance of using morphological descriptors in ecology to assess how communities interact, function, and respond within different environments. This review aims to provide a basic description of the mathematical principles underlying morphological quantifications.

## Introduction

The purpose of this review is to provide a common language from which biologists and mathematicians can begin a conversation on quantifying plant morphology. The idea was conceived from the National Institute for Mathematical and Biological Synthesis (NIMBioS) workshop on Plant Morphological Modeling. In this workshop, mathematicians and biologists came together to discuss how to advance the field of plant morphology. In small group discussions, we found that a substantial portion of our time was spent trying to understand what the other discipline meant when using the same words. In light of this, we decided to write a basic primer on the definition of common morphological quantifications and how they can be applied to plants. The goal of this review is to provide an introductory basis for further discussion and collaboration between math and biology.

## Ontologies for Plant Morphology

High quality morphological descriptions are critical for our understanding of biological systems, because often the appearance of a structure (e.g., leaf, cell, etc.) drives functionality (e.g., flux, nutrient transport, etc.). However, translating the visual appearance of complex organisms into qualitative and quantitative metrics remains a significant challenge in biology and mathematics. A major hurdle in advancing morphological studies is to find a common language for biologists and mathematicians to communicate. Ontologies are used to provide a common reference vocabulary (Planteome; Cooper and Jaiswal, 2016), and specific ontologies have been developed for plant structural descriptions (Plant Structural Ontologies; Ilic et al., 2007). However, these descriptions often focus on the biology and do not provide definitions for mathematical concepts. In this review we present our consensus on the definition and application of mathematical terms to describe the appearance of plant organs. While other interpretations can be considered, this review aims to provide a basic ontology of plant morphology to initiate interdisciplinary collaborations between biologists and mathematicians.

### Biological and Hierarchical Scales

Plants are complex systems and their morphology can be quantified at many different biological scales ranging from genes to organs to communities. In addition, there are hierarchical scales that encompass each of these biological scales in both space and time. Although morphology is generally quantified at a single biological scale, relating quantifications across scales is critical to improve our understanding of how morphology impacts physiology, growth, development, and ecology.

**FIGURE 1:**
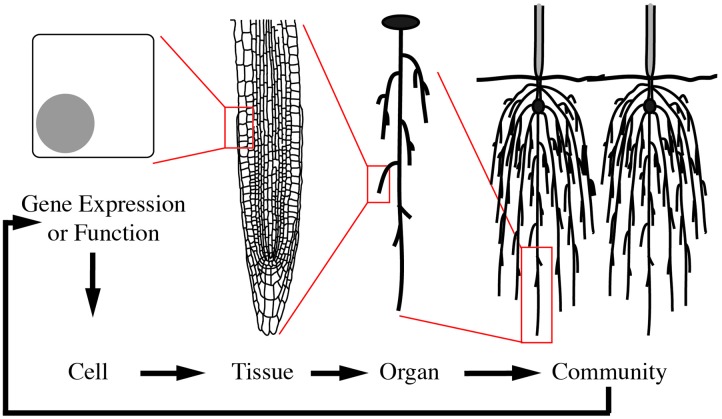
**Morphological changes are mediated through cyclic multi-scale signals and transformations**. A multi-scale morphology transformation is illustrated by neighbor detection between root systems. Morphological changes are initiated through developmental or environmental cues that induce changes in gene expression or function. These alterations lead to local changes at the cellular-level, which are then translated to the tissue- and organ-level. Organ-level changes then lead to an altered community. The community and environmental signals then feedback to mediate gene expression or functional changes in a continuous loop.

**FIGURE 2:**
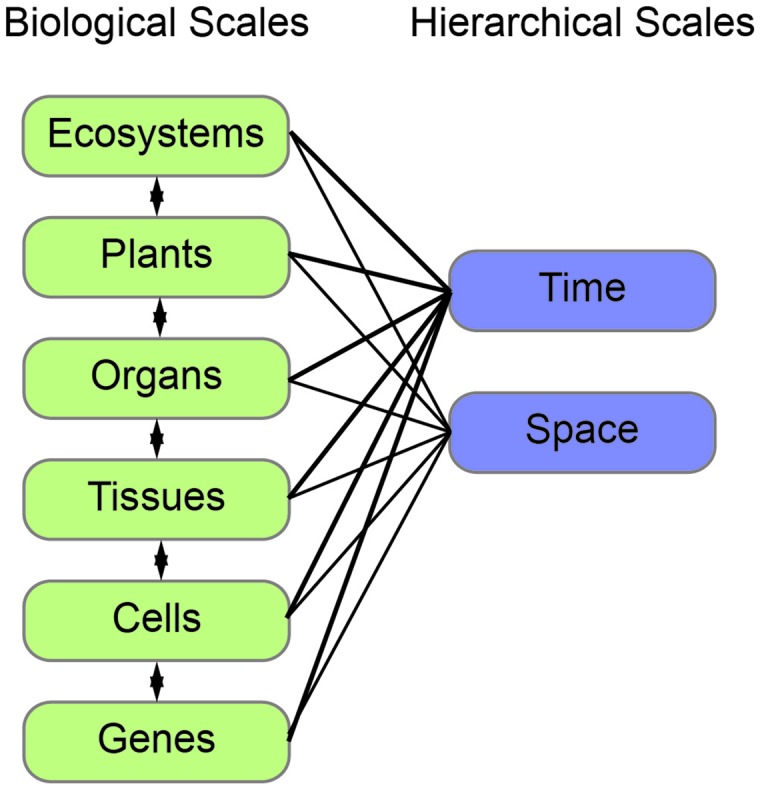
**The interconnection of biological and hierarchical scales**. Plant morphology can be measured and modeled at different biological (left, green) and hierarchical (right, blue) scales. Each of the biological scales influences the next and can be measured in both space and time.

To illustrate morphological transformations across biological scales, consider how neighboring root systems influence a plant community. In response to neighbors, a plant may alter root growth and development (Chen et al., 2012). This response is mediated through multi-scale signals and transformations (**Figure 1**). The process begins with changes at the gene expression or protein function level as a consequence of the genotype and/or external stimuli, in this case a neighboring plant. These alterations then lead to local changes at the cellular-level. For organ growth, the cell wall properties are altered to promote or inhibit expansion. For organ development, changes at the cellular level lead to cell divisions and influence the surrounding cells to enable new organ emergence. These cellular-level changes then mediate tissue-level changes, by communication with neighboring cells. Due to the constraints imposed by a cell wall in plants, one cell cannot grow or expand without support from its neighbors. The tissue-level changes then mediate organ level changes, which translate to the phenotype of the community. The community then relays signals back to the plant to mediate changes in gene expression or function and initiate the cycle again (**Figure 1**). This example is an over-simplification and in reality feedback occurs at each stage of the biological scales (**Figure 2**). Adding to the complexity, each of these biological scales can be analyzed on hierarchical scales of space and time (**Figure 2**). When designing an experiment that relies on plant morphological quantifications it is important to consider both the biological scale (from genes to ecosystems) and hierarchical scale. Later in this review we will provide specific examples at different biological scales.

### Image Acquisition and Data Reporting

Morphological measurements can be derived from different approaches including manual three-dimensional (3D) digitizing measurements (Sinoquet and Rivet, 1997; Godin et al., 1999) or image analyses (Li et al., 2014). We focus our discussion on image analysis approaches since manual measurements can be time consuming, low throughput, and subject to human error. Images can be generated in 2D or 3D, although both are generally expressed within a Cartesian coordinate system, denoted (x,y) and (x,y,z) respectively. In a 2D image, each discrete x,y coordinate is referred to as a pixel; similarly, in 3D the image coordinates are called voxels.

When considering the image acquisition system, it is first important to consider the scale of the process and resolution of the imaging system. For example, if a research question address tissue-level changes, it is likely that the resolution of digital microscopy is preferred to standard photography. In addition, the sampling frequency (static image versus time-series), physical scale (nanometers to meters), precision and accuracy required should be considered. Regardless of the image acquisition system (microscopy, X-ray tomography, photography, etc.) pre-processing is an essential first step. Pre-processing generally includes smoothing of the obtained imaging data to correct for noise of the imaging system. Once the noise has been reduced, the images can be used to extract morphological measurements. We refer the reader to the following resources for an in depth review on image processing techniques (Jain, 1989; Hartley and Zisserman, 2005), the available automation packages (Abramoff et al., 2004; Kuijken et al., 2015; Rousseau et al., 2015) and tools for computational reproducibility (Piccolo and Frampton, 2016).

After pre-processing, images may be analyzed with tools that are developed for a specific purpose or currently available. The online resource http://www.plant-image-analysis.org curates currently available tools for morphological plant image analysis and enables user feedback and ratings (Lobet et al., 2013). Regardless of the analysis, adhering to minimum standards for data reporting is vitally important for reproducibility. We emphasize the adoption of the Minimum Information About a Plant Phenotyping Experiment (MIAPPE; http://www.miappe.org/; Krajewski et al., 2015) standards.

### What is Shape?

As biologists, we often refer to the “shape” of an organ when we are describing morphology, however, this term can encompass a wide variety of mathematical parameters. Most often we are referring to the most intuitive quantification, geometry, which is used to establish measurable sizes of the plant organ surface. In this section we will define the mathematical tools that can be used to describe “shape” as a frame of reference for a biologist interested in morphological analyses.

**FIGURE 3:**
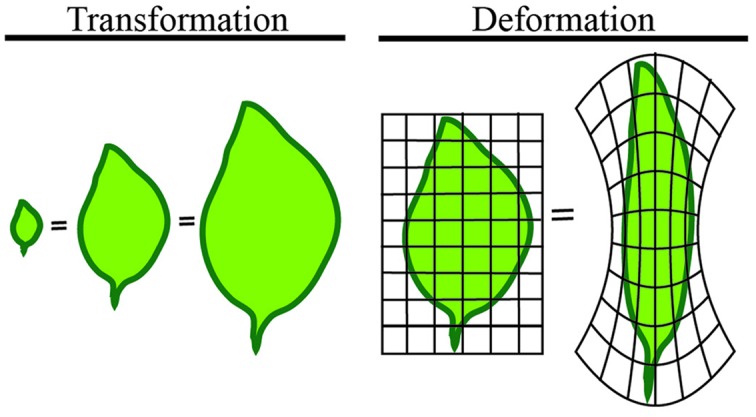
**Shape is independent of transformation or deformation**. The least intuitive quantification of morphology is shape. Shape refers to measures that are independent of transformation or deformation. The above leaves are all considered the same mathematical shape, despite dramatically different appearances.

We will first define shape in mathematical terms to provide a basis for the quantitative measures to follow. The mathematical concept of shape is difficult to represent because it differs from its geometric counterpart. A shape refers to the form of an object that may have several geometric representations, but an invariant associated topology (connections), and that makes measurements between features comparable. Comparable here means that shapes of the same type can be quantified independent of any transformation or deformation the shape undergoes (**Figure 3**). In the leaves illustrated in **Figure 3**, all are the same shape despite dramatically different appearances.

**FIGURE 4:**
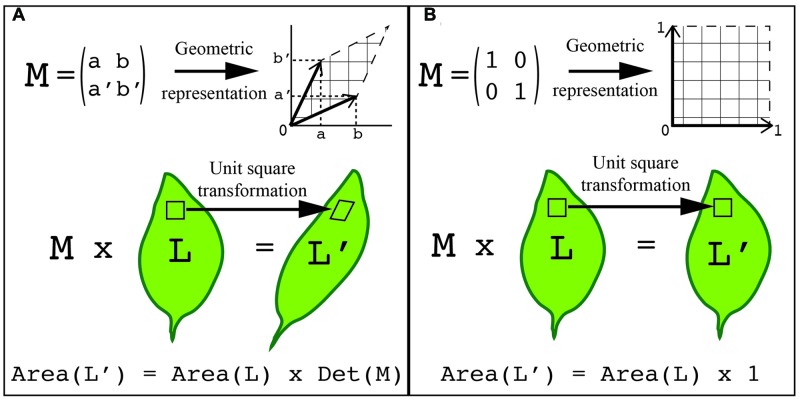
**Using transformations to quantify shape**. The points on the surface of an object can be represented with defined coordinates. These points can then be transformed by applying a transformation matrix. **(A)** A generalized representation of applying a transformation matrix to quantify shape. **(B)** An example of a unit transformation for shape changes. **(A,B)** In both examples, a 2x2 transformation matrix, M, can be visualized as a table of two column vectors (top left) or by geometric representation (top right). This same transformation can be visualized on a leaf. Each unit square that composes the organ surface can be transformed into the geometric shape associated with the transformation matrix (middle). The total area of the transformed organ is equal to the original leaf area times the transformation matrix determinant, which is the area formed by the two vectors (bottom). In the unit transformation matrix example, the matrix determinant, Det (M), is equal to 1.

One way to overcome this challenge is to represent the shape as a set of points on the surface of the object with defined coordinates. As the shape changes each point can be tracked through the transformation process. As a result, the transformation can be expressed with a matrix, and consecutive transformations can be expressed as the product of their matrices. Transformations inherit the geometrical and algebraic properties of matrices, which has the benefit that known rules of linear algebra apply. For instance, the determinant of a 2x2 matrix (where the determinant of matrix [a,b; c,d] = ad-bc) is equal to the area of the parallelogram defined by the column vectors of the matrix (**Figure 4**). Consequently, the area A′ of a shape transformed by a matrix M equals the area A of the original shape times the determinant of the matrix (i.e., A′ = A.det(M)). In this way, the application of two consecutive transformations represented by the two matrices M and M′ transform a shape of area A onto a shape of area A.det(M.M′) = A.det(M).det(M′). Another approach to describe a shape is to represent it as a basic geometrical model (sphere, cylinder, ruled surface, etc.). The repetition, union, or intersection of these basic building blocks can be used to describe more complex plant morphologies (Pradal et al., 2009). Later in this review, we describe how shape quantifications have been applied to gain an understanding of the genetic control of leaf morphology.

The mathematical definition of shape relies on two underlying quantifications – geometry and topology. While we will treat these quantifications as independent for the sake of simplicity, there is much overlap between these fields and the theoretical basis of each. Both approaches might characterize the same plant organ, but they refer to different types of quantifications that potentially lead to distinct biological interpretations. However, to truly understand the feedback between morphology and function, topological concepts should be viewed as complementary to the geometrical ones.

### Geometric Descriptors for Plant Morphology

**FIGURE 5:**
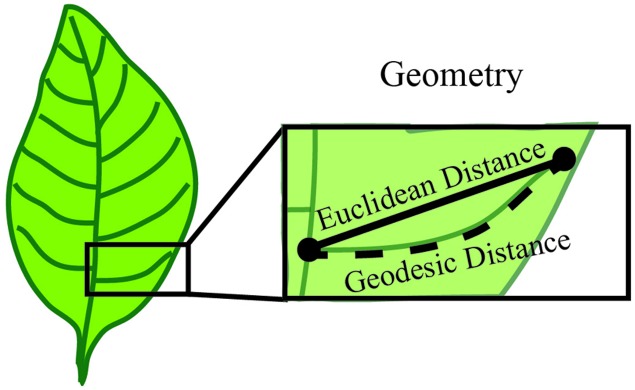
**Geometry establishes measurable sizes of the plant organ surface**. Geometry can be used to define parameters such as length, diameter and angle between features. In this example, the features are represented by the start and end of a vein branch (black dots). The distance between these two points can be described by the Euclidean distance, which is obtained by a straight line between two points. Alternatively, the shortest path along the branch surface between points, or Geodesic distance can be used to define length. Both of these are a valid measure of length and one common metric of geometry is the difference between these two measurements.

Geometry is used to establish measurable sizes of the plant organ surface. Basic geometric descriptors include vector, length, width, height, diameter, angle, surface and volume. For example, when considering the vein patterning of a simple leaf, the distance from a point of vein branch emergence to the branch tip is a geometric measure of length called the Euclidean distance (**Figure 5**). To overcome the limitations of basic geometric descriptors, compound descriptors computed from these basic descriptors can be considered for complex plant forms.

Compound descriptors include density, aspect ratio and spatial distribution, which can provide a general view of plant morphology. However, their use is often dependent on the resolution of the system used to image the plants. Further, the compound nature of these descriptors leads to a loss of information, which can limit the interpretation. Alternatively, derivative descriptors can be used to expand on the basic descriptors and provide metrics for complex morphologies.

Derivative descriptors include quantifying the border of an object, a curve, or a surface. These descriptors often leverage concepts from differential geometry such as curvature, torsion of a curve or the Gaussian curvatures of a surface. Quantification of patterns (e.g., symmetry and periodicity) can also be attained through derivative descriptors. To revisit the vein patterning of a simple leaf example, the basic Euclidean distance measurement of length underestimates the total vein length because it does not account for the curvature. Instead the length could be quantified as the shortest path along the branch surface from the point of emergence and the tip, termed the geodesic distance (**Figure 5**). In this instance, the geodesic distance provides a more accurate quantification of vein length because it traces the distance along the vein surface, which is not a straight line. Further, an estimate of vein curvature could be obtained from the difference of the Euclidean and geodesic distances. While derivative descriptors provide applicable metrics for plant morphology quantifications, the accuracy of these descriptors depends on precise and high-resolution image acquisition approaches.

Thus one approach to quantify morphology is through calculating one or more geometric descriptors. Later in this review, we describe how geometric descriptors have been and could be applied to understand cell growth and expansion. Regardless of the descriptors, it is vital to include which geometry is being used and the unit (e.g., inch or cm) of measure, because infinitely many metrics exist. Further, when reporting geometries that are derived from multiple measures it is critical to make the original data available. For example, a commonly used geometric measure is specific leaf area, which is the leaf area divided by the leaf dry mass. In this case, both the leaf area and the leaf dry mass should be reported. Further, if the geometric descriptors are covariant then reporting the mean geometric measures is not sufficient and individual measurements should be reported.

### Quantifying Plant Architecture with Topology

While descriptions of shape and geometry are often applied to individual plant organs, there is significant interest in defining how these organs are connected to generate plant architectures, which ultimately impact function. For example, the spatial arrangement of shoot branches directly influences the placement of leaves and has a significant impact on photosynthetic capacity (Rameau et al., 2015) This spatial arrangement is difficult to assess with geometry even if patterning (e.g., symmetry, periodicity) can be determined. Instead, other properties within the mathematical field of topology, such as connectivity, are essential to quantify the spatial arrangement of plant organs. Topology is defined as understanding how a property persists through geometric transformation and deformation of the object of interest. The simplest examples of topology utility are the description of branching structures (e.g., root system architecture or shoot branching architecture) and modeling connections between two objects (e.g., water flux between cells).

To understand the concept of connectedness between objects, it is important to first understand how a relationship is defined mathematically. A relationship is basically a rule that describes how elements of a set relate or interact with elements of another set. For example, a relation of connectedness (denoted by ‘∼’) between branches A and B only exists if a path along the plant surface links the two (i.e., A∼B). The order in which the elements are listed defines a strict relationship where A < B denotes the connectedness between user-defined reference points (e.g., tip of a branch to the base of the trunk). If we consider a tree crown, all tree branches can be ordered with regard to a defined reference point such as the point where the trunk emerges from the soil. However, if the tree is highly branched and has many ramifications, then it is often not possible to find a linear order to uniquely describe the tree crown. In this case, we can consider an additional relationship to denote the hierarchy of branches formed by the development program of the tree. If branch B emerges from branch A, we denote the emergence of a new level of branching hierarchy as A[+B]. Thus, considering the whole plant, two types of connections can be defined: an object A that precedes (type ‘A < B’) or bears (type ‘A[+B]’) a second object B (Godin and Caraglio, 1998; Godin, 2000).

**FIGURE 6:**
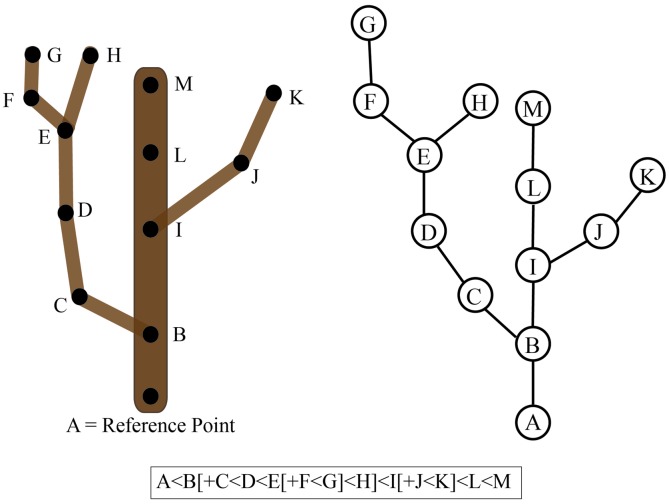
**Connectivity of a tree branch can be represented as a graph or a character chain**. Consider the tree on the left. The reference point was chosen as the base of this main branch (A). Each branch point is represented as a node (B-K) in the graph on the right relative to the reference point A. Alternatively, branch connectivity can be represented as a character chain, where the C < D relationship indicates that C is closer to the reference point (A) than D and B[+C] indicates that C is a child branch originating from the parent B.

These definitions of connectedness lead directly to the concept of graphs and of tree graphs. In mathematics, a graph is a representation of a set of objects where some objects are connected by links. Connected objects are represented by vertices (also called nodes or points), and the links that connect pairs of vertices are called edges (also called arcs or lines). Typically, a graph is depicted as a set of dots for the vertices, joined by lines or curves for the edges. Often nodes are referred to as “parents” and “children” based on the order of appearance. For example, if Node B is a main branch and Node A is a secondary branch originating from this main branch, then the parent of Node A is Node B. This can be represented as B < A or B[+A]. In this example, Node A is considered the child of Node B and Node B can have many children. Thus, connectivity can be represented as a graph or a character chain (**Figure 6**). If the graph has only one parent for each node, then it is considered a tree graph. Tree graphs are convenient to describe plant architecture, because a node can represent each branch and the relationship between branches can be represented as an edge of the graph. Such a connectivity graph is often termed a skeleton if the edges and vertices can be geometrically embedded into physical branching structure captured as imaging data (Bucksch et al., 2010; Bucksch, 2014).

**FIGURE 7:**
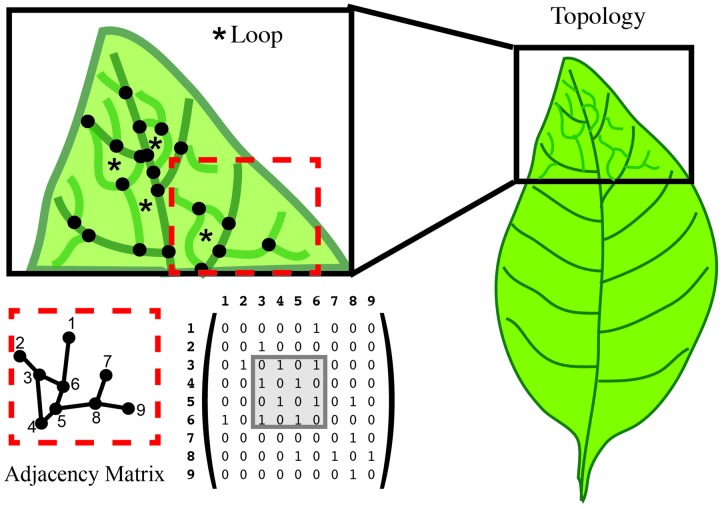
**Topology characterizes the relationship between features**. In this leaf vein example, features are defined as the junction of branches (black dots). Topology can calculate the number of loops (asterisk in the upper left), and the number of junctions forming that loop. An adjacency matrix that defines the connection between two features can be used to represent these data. The connection between features is represented in a numerical matrix. Connected features are represented by a “1” in the adjacency matrix. Loops can be visualized within the matrix (gray box).

One type of graph is defined such that every node is on a loop or cycle. Let us again consider the venation of a simple leaf. If we want to assess the complexity of the branched pattern, we can first represent the branch junctions as features (**Figure 7**). A relationship between junctions can exist if they are connected within the graph. Typical quantifications of such a topology can give the distance between junctions, the number of loops/cycles or the average number of junctions that form a loop/cycle (**Figure 7**). Topologies can also be represented independent of the physical organ with a graph that describes the adjacency of loops. One possible representation of the topological graph is adjacency matrix (**Figure 7**), which indicates the connection between junctions. Several types of information can be deduced directly from the adjacency matrix. For instance, two topological graphs are isomorphic if their matrices have the same minimal polynomial, characteristic polynomial, eigenvalues, and trace. The connectedness of topological graphs can also be deduced directly from the adjacency matrix (**Figure 7**).

In addition to representing topological properties such as connectedness, graphs facilitate the extraction of other properties, such as the distance from various reference points for each node. Additional properties make it possible to model complex and dynamic systems (e.g., nutrient transport or water flux) and enable multi-scale modeling. For example, multi-scale tree graphs (MTG) are used to describe tree structures at different scales (e.g., community, individual plant, plant branches, etc.). MTG is composed of a set of graphs, where a node in one graph (e.g., one plant within the community) corresponds to another graph representing another scale (e.g., the branching pattern within that plant). Another application for MTG is to model how nutrient or water flux between cells contributes to the whole organ physiology.

Lastly, one of the most successful approaches to modeling the development of plant architecture has been Lindenmayer (L)-systems (Lindenmayer, 1968; Prusinkiewicz, 1986). L-systems employ a recursive set of rules to grow branched systems (e.g., a grammar). An L-system model begins with an initial state from which to begin construction (called axiom), and a set of rules that define how each module (i.e., plant component) transforms over time. The model is then applied step by step to simulate geometrical and topological plant development. During the last 20 years, several implementations of L-systems have been designed: cpfg (Prusinkiewicz and Lindenmayer, 1990), L+C (Prusinkiewicz et al., 2007), XL (Hemmerling et al., 2008) and L-Py (Boudon et al., 2012), among others. Each L-System language provides a dedicated modeling language that mixes classical programming languages (e.g., C, C++, Java, Python) with mathematical notation based on formal language theory. These languages have also emerged to take into account the increasing complexity of the developmental models. Some variants of the initial formalism are stochastic L-Systems (Eichhorst and Savitch, 1980), environmentally sensitive L-Systems (Prusinkiewicz et al., 2007) and relational growth grammars (Kurth et al., 2005). Independently, Godin and Caraglio (Godin and Caraglio, 1998) have introduced a multiscale formalism, the MTG, to be able to encode any type of plant architecture data at different spatial and temporal scales. While a large community has adopted this formalism, the use of multiscale modeling to simulate the dynamic development of plants is quite recent (Boudon et al., 2012; Ong et al., 2014).

### Quantification of Changing Morphology

There is a substantial effort to assess morphology in relation to physical processes (e.g., response to biotic and abiotic environments or growth). Thus, we outline the basic concepts of comparative morphology in this section. The comparison of shapes is central to morphological quantification, and two mathematical principles exist to compare shapes. The first, reduction, relies on the removal of unimportant features and the merging of similar or equivalent features into a simplified “map.” Given a set of plant organs, the implication is that there is an established map to perform abstraction for all plant organs. Similarly, a distinct map exists for each plant organ that will recover the original details from the reduction. Reduction stores and quantifies the difference between a given organ shape and the abstraction of the plant organ shape. The second principle, registration, reorganizes feature locations such that comparable features of two plants are aligned to the same coordinate system to minimize the distance between the features. While these principles can be applied for comparison between two objects (e.g., plants or organs), they can also be used to compare objects to a reference geometrical object modeling the shape (e.g., comparing fruit shape to a sphere). For example, given a set of leaves with different shapes, shapes can first be registered and a mean shape computed. Then for each leaf, the reduction map between this leaf and the mean leaf (abstraction) can be calculated. This map will then quantify the distance between a shape and its reduction, and provide a metric of similarity.

Measuring changes in geometry is relatively straightforward because geometry derives measureable terms. In the leaf vein example, one could compare average length of veins or vein diameter between two samples. It is important to use comparable metrics within these comparisons. For example, the Euclidean length from one sample cannot be directly compared to the geodesic length of another sample. Again with geometric quantifications it is important to indicate which metrics are being used and the units of measure.

For basic topological quantification, such as number of loops or number of junctions forming a loop, straightforward comparison can be made. However, when considering the dynamics of changing topologies, the adjacency matrices can be very useful (**Figure 7**). In an adjacency matrix, each position corresponds to the intersection of two junctions or nodes. Directly comparing adjacency matrices relies on the same order of nodes referenced at each position in the matrix. Thus, adjacency matrices are most useful for the quantification of changes within a single system. For example, when quantifying the development of veins within a leaf, node definitions are generated based on the final topology and traced back in time. This will generate equal size adjacency matrices for comparison across the development and enable quantification of new (birth-rate) and lost (death-rate) topologies at any given time. Matrix algebra applications can be applied to these matrices for comparison between plants. If instead, topological comparisons are required between systems where the nodes differ, then network alignment algorithms can be utilized. These algorithms attempt to maximize the topological similarity between two different networks, and are most frequently applied to gene-based networks (e.g., Kuchaiev et al., 2010; Milenkovic et al., 2010; Ficklin and Feltus, 2011). However, the current implementations are fraught with technical problems and the choice of algorithm can influence the outcome (e.g., Clark and Kalita, 2014). While not extensively used for morphological assays, these comparison tools can be adapted for evolutionary, cross-species, or interspecies comparisons. In the remainder of the review, we will highlight some examples of how mathematical descriptors have been and can be applied to quantify morphology in biological systems.

### Leaf Morphological Traits

We have utilized a leaf in the above illustrations, because morphological quantifications have been applied most extensively in this area. Leaf morphology is directly tied to various functions, including water uptake (e.g., Ito et al., 2015), photosynthetic capacity (e.g., Reich et al., 1998) and gas exchange (e.g., de Boer et al., 2016). However, leaf morphology is dynamic and changes in response to the environment (Chitwood and Sinha, 2016). Thus, there is an effort to quantify leaf morphology over time to lend a broader understanding into leaf function.

Two of the most common metrics for quantifying leaf morphology are the geometric measures of leaf area and specific leaf area (Kleyer et al., 2008). Leaf area is calculated as the surface area of one side of a single leaf, generally expressed as mmˆ2. While specific leaf area, as mentioned above, is the leaf area divided by the leaf dry mass, generally expressed as mmˆ2/mg (Kleyer et al., 2008). The differential use of these two metrics throughout the literature highlights the importance of reporting the original data and the geometric measure applied.

Basic geometric measures of length and width have also been applied to quantify changes in maize leaf shape, which have stereotypical linear or linear-lanceolate (pointed at both ends) leaves (Tian et al., 2011). However, for leaf shapes of increased complexity (e.g., palmate, pinnate, lobed, etc.), more sophisticated analyses have been applied. One approach, which can be applied to the same leaf changing over time or different leaves with the same type of classification (e.g., number of lobes), relies on leaf shape homology. In this approach, the same number of points are placed equidistant along the curved edge and expressed within a Cartesian coordinate system. Anchor points are defined by homologous features between leaves (e.g., base and tip) to facilitate alignment of the coordinate systems. Dimension reduction techniques (e.g., principal component analysis) are then applied to identify the points that most efficiently explain the differences in shape between the leaves (Feng et al., 2009). This approach has been successfully used to identify the genetic basis of leaf and petal shape and size in snapdragons (Langlade et al., 2005; Feng et al., 2009; Cui et al., 2010) and to characterize the diversity and effect of climate on grape leaf morphology (Chitwood et al., 2016a,b).

An alternative approach views the leaf shape as a closed contour formed by a wave connecting back on itself. This approach is particularly suited for the comparison of leaves without homologous points. This analysis begins by converting the shape into a numeric vector called chain code, which defines a contour as a series of linear fits (Kuhl and Giardina, 1982). The chain code vector is then used to calculate Elliptical Fourier Descriptors. In the simplest terms a Fourier transform fits a series of sine waves to an object. In this case, the Elliptical Fourier Descriptor takes the Fourier transform of the boundary of the object within a closed elliptical. The Fourier transform is run at multiple Fourier coefficients to produce harmonics. The more harmonics that are utilized, the greater the complexity of the resultant curve. This approach has been successfully used to identify the genetic basis of tomato (Chitwood et al., 2012, 2013) and grape (Chitwood et al., 2014) leaf morphology.

### Modeling Cell Growth and Expansion

Changes at the cellular-level are an important mechanism by which plants grow and develop. In this section we will discuss two biological systems, the hypocotyl and floral sepals, which have been utilized to understand how growth and expansion contributes to organ morphology. The hypocotyl has been used to study cellular expansion since the mid-1800s, however, the floral sepal system is a more recently developed model.

**FIGURE 8:**
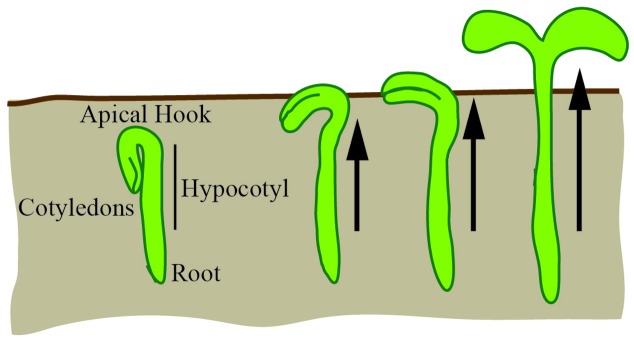
**The hypocotyl is a model for cellular expansion and growth**. In the dark, under the soil, the hypocotyl forms an apical hook. As the hypocotyl expands and grows toward the light, the hook expands to unfurl the cotyledons. Hypocotyl growth is driven by unidirectional cellular expansion (arrows).

The hypocotyl is the stem of a germinating seed that connects the cotyledons and the roots in eudicot plants. In the dark, the hypocotyl forms a hook (apical hook) that is believed to protect the cotyledons from damage as the seedling navigates through the soil; the hook subsequently opens when the seedling is exposed to light (**Figure 8**). Most of the hypocotyl growth in the dark derives from cell expansion primarily driven by the outer epidermal cells (Gendreau et al., 1997; Savaldi-Goldstein et al., 2007). For a cell to expand, it must balance the requirements for structural support and elasticity. At the basic level, this requires a balance between the cytoskeletal structural components and the loosening and synthesis of cell wall components (reviewed in Bashline et al., 2014). In the hypocotyl, expansion occurs along the longitudinal axis with cortical microtubules limiting cell expansion to this axis and spatially controlling cellulose synthesis (reviewed in Vandenbussche et al., 2005). Since growth of the hypocotyl is predominantly due to unidirectional cell expansion, it is an ideal system to apply basic geometric descriptors to study the control of growth.

In its simplest form, the hypocotyl (excluding the apical hook) can be viewed as a cylinder that is hydrostatically uniform and with a radial water potential gradient (Kutschera and Niklas, 2007). The rate and extent of cylinder expansion is controlled by genetic and environmental factors. Hypocotyl elongation has been primarily modeled independently of cellular morphology as an outcome of kinetic parameters written as a set of ordinary differential equations (Chew et al., 2014). It is unclear how individual cell expansions (as quantitated by geometric or shape descriptors) lead to the collective growth of the hypocotyl and contribute to differential growth. For example, there is little known about how the apical hook is formed, maintained and opened during elongation. Additionally, nutational bending as a result of differential growth is observed, but the mechanism(s) for this are still not fully understood. These open questions could be targeted through time-series image acquisition and a temporal analysis of geometric descriptors (e.g., the aspect ratio of each cell in the hypocotyl over time). The goal of such an analysis is to identify emergent properties that could explain experimental observations (e.g., nutational bending). Thus, even in a simple and well-studied system such as the hypocotyl, the quantification of differential cell morphology has the potential to contribute to our understanding of growth control.

A more recently established system to study complex growth control is the flower sepals. In complex organs overall morphological structure is crucial for their proper function, while morphogenesis is a dynamic process in which the topology and geometry change over time. The establishment and maintenance of proper shape and size is a fundamental developmental process of all multicellular organisms, but how it is tightly regulated remains a mystery (Vogel, 2013). *Arabidopsis thaliana* sepals are used as a model to study this process because of their accessibility for live imaging and cellular growth analysis (Roeder et al., 2010, 2012; Qu et al., 2014; Tauriello et al., 2015; Hervieux et al., 2016; Hong et al., 2016).

**FIGURE 9:**
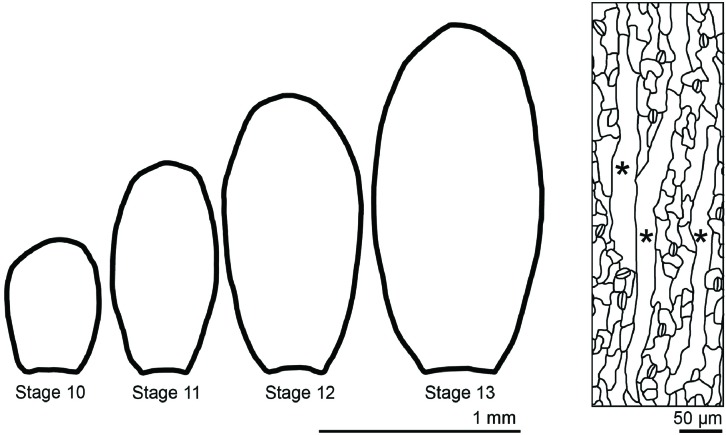
**The morphology of sepals and their epidermal cells**. Wild-type *Arabidopsis thaliana* sepals decrease the width/length ratio as they grow. Sepal epidermal cells are highly variable in morphology, with giant pavement cells (^∗^) interspersed between smaller cells in a large range of sizes.

Sepals are the outermost sterile organs of a flower, which surround and protect the developing reproductive structures inside the bud before the flower opens. The sepals start from the small dome-shaped sepal primordia initiating from a line of eight cells on the edges of the floral meristem (Bossinger and Smyth, 1996). The young sepals grow in both medial-lateral and proximal-distal directions, maintaining a relatively low aspect ratio. Gradually, the sepals grow more in the proximal-distal direction, leading to increased aspect ratio (**Figure 9**). Mature sepals are roughly elliptical, approximately 2 mm long, 1 mm wide, but less than 50 μm thick. Therefore, they are considered flat organs, and 2D geometric descriptors such as length, aspect ratio, and circularity can be used to describe their morphology.

Similar to hypocotyl growth, epidermal cells largely control sepal growth. The morphology of epidermal cells affects the overall sepal curvature and sepal shape. Through the application of a combination of quantitative and qualitative geometric descriptors, it has been shown that the abaxial sepal epidermal cells display a wide distribution of size and shape (Roeder et al., 2010). Giant pavement cells have an area up to 20,000 μm^2^, but are generally long and skinny, with high aspect ratios, whereas the smallest cells have an area less than 100 μm^2^ and are more round with high circularity. These small cells can be quite irregular in shape, though they are generally less interdigitated than leaf pavement cells (**Figure 9**). As described above, mathematical methods such as principal component analysis and elliptical Fourier analysis have been used to describe organ shape and size (Bensmihen et al., 2008; Chitwood et al., 2013). With these approaches, a more comprehensive description of morphological change in cells and organs integrating with genetics will facilitate the understanding of the underlying mechanism of shape determination.

Besides the morphological variability, sepal pavement cells display variable growth and division as well (Roeder et al., 2010; Schiessl et al., 2014; Tauriello et al., 2015). Principal directions of growth, growth isotropism and areal increase have been used to analyze cellular growth pattern. Similar to leaves (Beemster et al., 2005), sepals have a gradient of cell growth. Young sepals have fast and anisotropic cell growth at the tip while the growth in the lower sepals is slower and more isotropic. As the sepal grows, cell areal growth rate, growth anisotropy and cell division rate progressively decrease from the tip downward (Hervieux et al., 2016). Sepal morphology also varies across different development stages, environmental conditions, and in different genetic backgrounds. Despite high variability on the cellular level, sepals require coordinated growth to form an effective barrier to protect the meristem (Hong et al., 2016). A comprehensive analysis of morphology on the organ and cellular levels can bring insights into sepal function and the mechanisms regulating morphological diversity.

### Quantifying Root System Architecture

Root system architecture (RSA), which describes the spatial configuration of different types of roots in the root system (Lynch, 1995), is integral to water and nutrient uptake. Because of this, research has focused on imaging and quantifying RSA. Key questions include: ‘What genes underlie particular root traits (e.g., deep roots or wide root systems) and how do they function?” “What root traits and architectures are optimal for specific environments?” “What are the functions of different types of roots and how do they contribute to the function of the entire root system?” Knowledge gained from addressing these questions will enhance the ability of plant breeders to develop crops with robust root systems that lead to increased crop production in harsh environments.

Root system architecture includes the topology of the root system, which describes the network and pattern of root branches (Berntson, 1997) as well as the distribution of roots, which refers to the presence of roots within a given region (Lynch, 1995; Jung and McCouch, 2013). Historically, finer features of the root system, such as root hairs, were not included in RSA (Lynch, 1995). However, more recent definitions of RSA embrace multiple scales (Smith and De Smet, 2012; Jung and McCouch, 2013; Lobet et al., 2015) and include both macroscale and microscale features such as root hairs and root diameter. Root anatomy, the internal cellular organization of the root, is not generally considered part of RSA, but recent work suggests it affects RSA (Zhu et al., 2010; Postma and Lynch, 2011; Jaramillo et al., 2013; Lynch et al., 2014; Saengwilai et al., 2014). RSA is generally divided into two broad classes, a taproot system found in most dicots and a more complex RSA found in many grass species that consists of a bushier root system with different types of roots, including shoot-borne roots.

By configuring the spatial distribution and network of roots within the soil, RSA significantly impacts the ability of roots to function in water and nutrient uptake. RSA is highly responsive to environmental signals, allowing the root system to adapt to different soil environments (Hodge, 2004; Lynch, 2011, 2013, 2014; Smith and De Smet, 2012). For example, phosphorus is concentrated in the topsoil. Thus, bean varieties more adapted to phosphate deficiency have shallower root systems, increased lateral roots in the upper region of the root system, and increased root hair density to maximize phosphate uptake (Bonser et al., 1996; Lynch and Brown, 2001; Lynch, 2011; Peret et al., 2014; Miguel et al., 2015). Work with nitrate has provided evidence of both local and systemic nitrogen signals that impact root growth. For example, a split root experiment showed that local patches of high nitrate elicit lateral root outgrowth while root systems grown in conditions of globally high nitrate have fewer elongated laterals (Ruffel et al., 2011). In contrast, globally deficient levels of nitrate substantially increased root growth and branching, but locally deficient levels did not (Zhang et al., 1999; Ruffel et al., 2011).

Root system architecture is a complex trait controlled by a small contribution from many genes (Topp et al., 2013; Zurek et al., 2015). Due to the importance of RSA in plant growth, fitness, and defense, a major goal is to identify the genes underlying specific RSA traits. Identification of these genes requires the ability to accurately quantify the trait of interest, which in turn necessitates the ability to image and analyze root system morphology. Recent years have seen an explosion in the development of both root architecture imaging technologies (Iyer-Pascuzzi et al., 2010; Clark et al., 2011; Mairhofer et al., 2013; Rellán-Álvarez et al., 2015), and the software for quantifying RSA (Armengaud et al., 2009; French et al., 2009; Lobet et al., 2011; Galkovskyi et al., 2012; Mairhofer et al., 2012; Bucksch et al., 2014; Das et al., 2015). In addition, modeling approaches, which combine RSA under different environments, have been used to successfully predict the optimal RSA, or ideotype for specific environments (Lynch, 2007; Draye et al., 2010; Leitner et al., 2010; Pages, 2014).

Each of the growth and imaging technologies for RSA quantification has its own pros and cons (reviewed in Piñeros et al., 2016). These technologies can be destructive or non-destructive, and range from simple and inexpensive to complex and expensive. Image quantification can occur in 2D or 3D. Frequently used, more inexpensive, and non-destructive technologies include growth in gellan gum or agar (Iyer-Pascuzzi et al., 2010; Clark et al., 2011; Fang et al., 2013) or in pouches (Hund et al., 2009). In these simple systems, roots can be imaged either by scanning or imaging with a digital camera, and analyzed with any number of software packages (see again http://www.plant-image-analysis.org for an overview; Lobet et al., 2013). Although these systems enable inexpensive and non-destructive quantification of RSA traits, the roots are not grown in soil, which may limit the application of these results in a natural setting.

The plasticity of RSA in different environments has led to significant interest in the development of non-destructive imaging technologies that image roots grown in soil or potting mix. One such technology grows roots between two plates in soil and relies on luminescence-based reporters for visualization (Rellán-Álvarez et al., 2015). While this technology is perhaps ideal for inexpensive and non-destructive imaging of roots in soil, it is limited by the requirement for transgenic reporter plants.

An alternative approach for imaging soil-grown plants non-destructively is X-ray computed tomography (X-ray CT; Mairhofer et al., 2013). In this technique, plants are grown in soil in pots and can be imaged daily. Rates of RSA growth and the response to environmental conditions can be observed over time, and the images can be reconstructed in 3D, which increases accuracy. Although more expensive and data-intensive, by imaging roots grown in soil, this technology promises to yield information more applicable to natural settings. One drawback of X-ray CT is the expense and the current inability to implement it in the field. In contrast, a straightforward, albeit destructive, method of field-based RSA phenotyping overcomes both of these obstacles. Termed ‘shovelomics,’ this method examines only the upper region of the root system that can be removed from the soil without much damage (Trachsel et al., 2011). Roots are washed of soil, images taken in 2D, and root traits quantified with available software packages, such as the “Digital imaging of root traits” or DIRT package (Bucksch et al., 2014; Das et al., 2015). DIRT uses an imaging pipeline to extract basic geometric root traits such as length, angle and diameter from the underlying graph representation, as well as novel descriptors of shape deformation. Recently, these descriptors were used to identify shape properties of cowpea roots that are associated with Striga tolerance (Burridge et al., 2016).

The ability to image, quantify, and model RSA is leading to new discoveries regarding the genes and genomic regions that control these complex traits (Topp et al., 2013; Zurek et al., 2015). Additionally, these technologies allow key questions across scales to be addressed. One such question is how (or whether) microscale features of RSA such as root hairs and root diameter impact macroscale features of RSA such as root branching. For example, root cortical aerenchyma (RCA) is open space in the root formed from the cell death of root cortex. By no longer requiring nutrients and carbon, RCA may alter the metabolic cost of root architecture such that roots can grow deeper or thicker (Lynch, 2013, 2014). SimRoot (Lynch et al., 1997) is a structural–functional model that emerged from a large amount of empirically collected data. Additionally, SimRoot incorporates physiological models of nutrient and water uptake. Software such as SimRoot can model changes in RCA and make prediction on the effects of competition in root architecture.

A recent developed software package, DynamicRoots, merges geometric and topological approaches to quantify the growth of a root system (Symonova et al., 2015). In this package 3D reconstructions of time-series RSA are first registered to the same coordinate system. These images are then converted to a series of graphs with nodes representing the voxels and edges representing the connections between neighboring voxels. Several calculations can now be derived from these graphs. For example, the addition of new edges that persist likely indicates a new branch forming and the geodesic distance between a reference point and each voxel can be used to track tip growth. Using these types of information, DynamicRoots can decompose the RSA into individual branches and extract branch-specific geometries (Symonova et al., 2015). This software is the first aimed at analyzing growing root systems from time-series data and has the potential to provide key insights into the local and global RSA impacts of changing environmental conditions.

### Morphology as a Tool for Ecology

All of the analyses highlighted above are important to contribute to our understanding of how whole communities respond, function, and interact within a particular environment. Recently, plant trait-based ecology has pushed forward many basic ecological questions. For example, quantifying plant traits helps understand variation in trait function across ecological scales (e.g., Auger and Shipley, 2012; Verheijen et al., 2013), relative importance of intra- versus inter-specific trait variation across these scales (e.g., Violle et al., 2012; Kichenin et al., 2013; Siefert et al., 2015), community assembly processes (e.g., Hille Ris Lambers et al., 2012; Laughlin and Laughlin, 2013), and eco-evolutionary dynamics (e.g., Vellend and Geber, 2005; Whitham et al., 2006; Hughes et al., 2008; Bailey et al., 2009a,b). Above- and below-ground plant traits such as leaf area and shape, root formation, and above- to below-ground biomass ratios are morphological traits that allow ecologists to make predictions about plant ecological strategies and overall community response to environmental changes (Suding et al., 2008). While genetic variation has been shown to have consequences on community and ecosystem function (e.g., Bangert et al., 2006; Crutsinger et al., 2006; Johnson et al., 2006; Lankau and Strauss, 2007), directly connecting genotypic diversity to phenotypic variation for ecologically important traits has been more difficult. The proliferation of open-source, global plant trait databases (e.g., TRY plant trait database: https://www.try-db.org/TryWeb/Home.php, Glopnet: http://bio.mq.edu.au/∼iwright/glopian.htm) as well as large-scale plant phenotype databases (unPAK: http://arabidopsis.biology.cofc.edu/) allows plant ecologists and evolutionary biologists to quickly harness trait data to answer relevant ecological and evolutionary questions. Building these databases so that they incorporate trait data relevant across multiple scales and environments is essential to providing a holistic view of plant-environment and plant-plant interactions. Essential to the building of these databases is the rapid quantification of the plant morphological traits through the application of mathematical principles.

The establishment of larger and standardized plant trait databases would provide ecologists with the necessary amount of data to gain insight into linkages between genotype and phenotype, and the potential impact on community and ecosystem processes. Quantifying changing leaf morphology would provide insights into physiology through easier-to-measure traits such as specific leaf area. Quantifying changing topology of root formation and architecture could provide insight into drought tolerance at a single time point and eventually over time. In addition, quantifying the dynamic topology of stem elongation and branching patterns could provide insight into light acquisition under changing light environments. Finally, morphological models for above- and below-ground traits could be integrated to understand the potential links and tradeoffs under different environmental conditions. For example, these models could be instrumental in understanding tradeoffs in resource allocation between roots and shoots, particularly in harsh conditions. Tradeoffs between above- and below-ground structures would likely differ across a light versus soil resource gradient. Together, these morphological models have the potential to help us make connections between plant genetics, morphology, physiology, and ecosystem dynamics.

## Conclusion

In this review we have outlined the basic principles of morphological quantification in terms of geometry, topology and shape. The choice of mathematical descriptor(s) and analysis depends heavily on the biological and hierarchical scale. We have highlighted a few examples of morphological descriptors that have been applied across biological scales. For both cellular growth and organ morphology, basic geometric descriptors and/or shape analyses can be applied to extract traits relevant to genetic, environmental, and evolutionary diversity. While these same principles can also be applied to root systems, there is an added advantage to using topological measures to quantify how individual components are connected within the whole structure. Lastly, each of these individual analyses provides important insight into the larger context of how plants function within a community. The advancement of plant morphological quantification and the interdisciplinary collaboration between biologists and mathematicians is critical to elevate our understanding of plant development, function, and evolution. It is our hope that this review will encourage more interdisciplinary interactions and promote research in the field of plant morphological modeling.

## Author Contributions

Authors are listed in alphabetical order and all authors contributed equally to writing and editing the manuscript. EES coordinated and integrated individual contributions.

## Conflict of Interest Statement

The authors declare that the research was conducted in the absence of any commercial or financial relationships that could be construed as a potential conflict of interest.
